# Is Pulp Inflammation a Prerequisite for Pulp Healing and Regeneration?

**DOI:** 10.1155/2015/347649

**Published:** 2015-10-11

**Authors:** Michel Goldberg, Akram Njeh, Emel Uzunoglu

**Affiliations:** INSERM UMR-S 1124 & Université Paris Descartes, Sorbonne, Paris Cité, 45 rue des Saints Pères, 75270 Paris Cedex 06, France

## Abstract

The importance of inflammation has been underestimated in pulpal healing, and in the past, it has been considered only as an undesirable effect. Associated with moderate inflammation, necrosis includes pyroptosis, apoptosis, and nemosis. There are now evidences that inflammation is a prerequisite for pulp healing, with series of events ahead of regeneration. Immunocompetent cells are recruited in the apical part. They slide along the root and migrate toward the crown. Due to the high alkalinity of the capping agent, pulp cells display mild inflammation, proliferate, and increase in number and size and initiate mineralization. Pulp fibroblasts become odontoblast-like cells producing type I collagen, alkaline phosphatase, and SPARC/osteonectin. Molecules of the SIBLING family, matrix metalloproteinases, and vascular and nerve mediators are also implicated in the formation of a reparative dentinal bridge, osteo/orthodentin closing the pulp exposure. Beneath a calciotraumatic line, a thin layer identified as reactionary dentin underlines the periphery of the pulp chamber. Inflammatory and/or noninflammatory processes contribute to produce a reparative dentinal bridge closing the pulp exposure, with minute canaliculi and large tunnel defects. Depending on the form and severity of the inflammatory and noninflammatory processes, and according to the capping agent, pulp reactions are induced specifically.

## 1. Introduction

**Figure 1:**
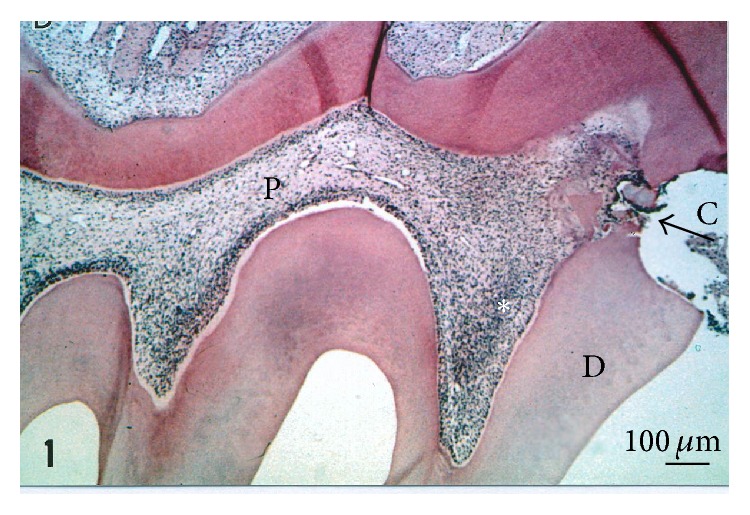
Pulp exposure and moderate inflammatory process. A cavity (C) was drilled on the mesial aspect of the six-week-old rat's first maxillary molar. One week after the pulp exposure, dentin debris is pushed in the pulp exposure. A moderate inflammatory reaction is seen in the mesial pulp horn (white asterisk). Hematoxylin-eosin staining. P = pulp. * *Bar = 100 *μ*m.

The alternative stages of dental pulp inflammation were restricted for many years with two limited options: necrosis or apoptosis. They appeared to be closely associated with four cardinal signs, reported in many references found in the literature as rubber, dolor, color, and tumor (swelling). Several small molecules and proteins are normally kept within the cells. In these areas, extensive cell death and tissue necrosis, also called coagulation necrosis, may also occur. More recently, a cascade of four stages was identified. There is actually a need for redefinitions of the physiopathological events, which might occur. The dental pulp may be exposed to the carious lesion or influenced by the adverse effects of filling materials (Figure 1). The inflammatory processes are gradually increasing from mild (moderate) to severe inflammation. Subjected to necrosis or apoptosis, nemosis has been recently added to the list of processes implicated in the destruction of the dental pulp [1, 2]. Pulp healing is the first step, followed by regeneration. This cascade of events is directly linked to the deleterious effects of inflammation processes in the presence or absence of pulp remnants.

**Figure 2:**
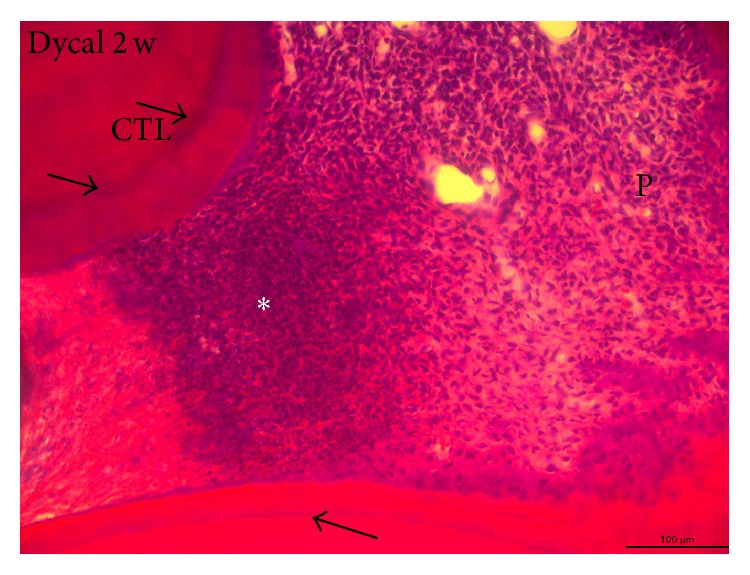
Pulp capping with calcium hydroxide (Dycal). Two weeks after the direct capping of a calcium hydroxide (Dycal), within the pulp calciotraumatic lines (CTL) (arrows), separate the dentin formed before the preparation of the cavity from the reactionary dentin (RD). In the left part, the Ca(OH)_2_ has induced the formation of a reparative bridge in the isthmus separating the central from the distal pulp horns of 6-week-old rat's maxillary molar. On the left part of the pulp, the necrotic tissue is acellular, whereas in the right part of pulp, the vital pulp displays proliferating inflammatory cells (white asterisk). In the right part of the figure, pulp cells (P) differentiate and form odontoblast-like cells. * *Alizarin red staining. Bar = 100 *μ*m.

The repair of dental pulp by direct capping with calcium hydroxide [Ca(OH)_2_] or by implantation of bioactive extracellular matrix (ECM) molecules implies four sequential steps: a moderate inflammation, the commitment of adult reserve stem cells, their proliferation, and terminal differentiation [3] (Figure 2). Most of the published studies report that the healing sequence starts with an initial moderate inflammatory process, and now there are evidences that inflammation is a prerequisite for tissue healing as a first step, followed by pulp regeneration, also described as pulp repair.

## 2. Mechanisms Implicated in Pulp Inflammation

### 2.1. Inflammation**

**Figure 3:**
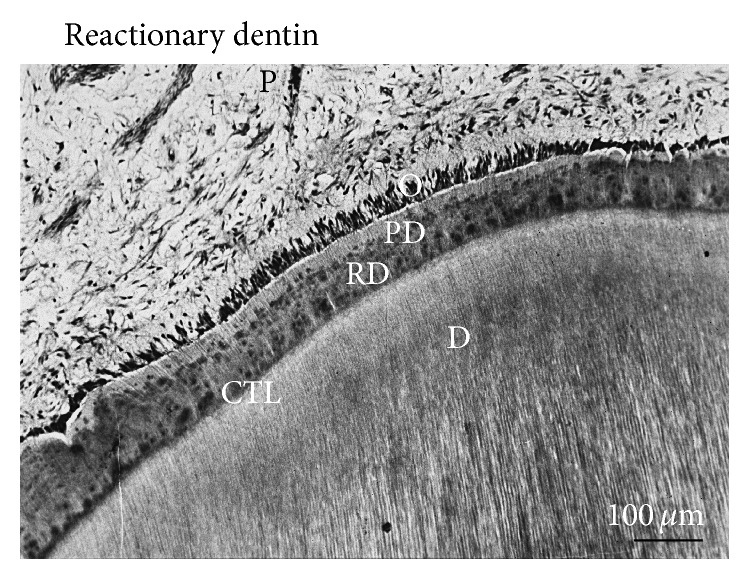
Reactionary dentin formation beneath a calciotraumatic line. A calciotraumatic line (CTL) separates the tubular secondary dentin (D) from the reactionary dentin (RD) formed in response to the treatment of carious lesion of human premolar (young adult). Odontoblasts (O) located in the outer layer of the pulp (P) synthetize and secrete the components of predentin (PD). Hematoxylin-eosin staining. P: pulp. * *Bar = 100 *μ*m.

**Figure 4:**
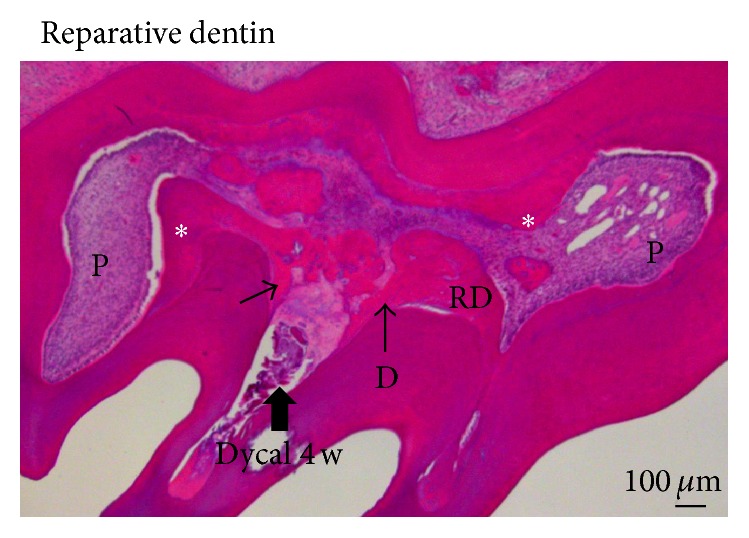
Formation of a reparative dentinal bridge. Six-week-old rat's first maxillary molar, followed by pulp capping with Dycal, 4 weeks after Ca(OH)_2_ implantation within the pulp exposure (thick arrow). The dentinal reparative bridge (arrows) is still incomplete. Tunnels and other defects connect the oral cavity and the dental pulp. Along the walls of the pulp chamber, a dense and continuous layer of reactionary dentin (RD and white asterisks) is formed, reducing the pulp (P) volume. Hematoxylin-eosin staining. Bar = 100 *μ*m.

**Figure 5:**
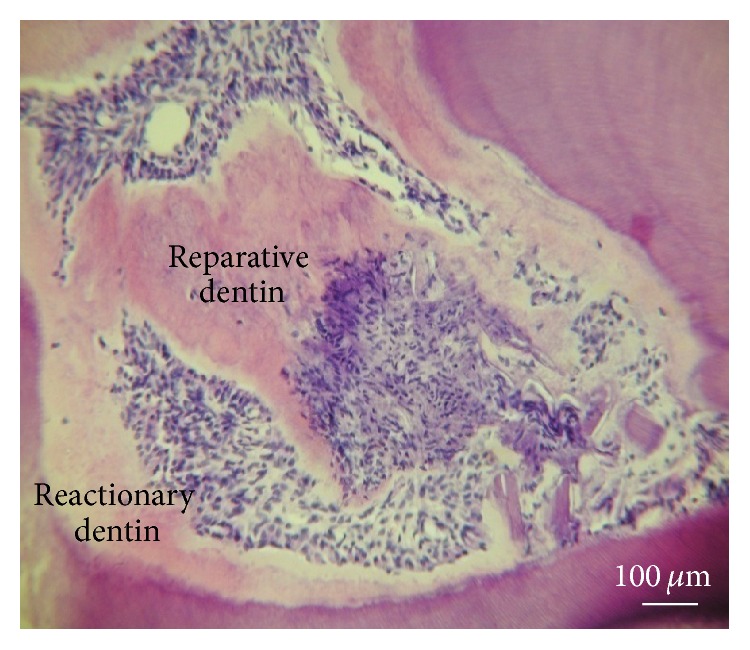
Reparative and reactionary dentin formation. After filling a cavity prepared in the mesial aspect of the rat's first maxillary molar, with Biodentine, a Ca_2_SiO_3_-based restorative cement, an early inflammatory reaction was stimulated. Inflammatory reaction of pulp cells producing reactionary (reduction of pulp volume) and reparative (formed in the center of the pulp volume) dentins. Hematoxylin-eosin staining. * *Bar = 100 *μ*m.

The importance of inflammation in pulp healing has been underestimated, for a long time considered only as an undesirable effect, leading in most cases to pulp necrosis and other adverse consequences. In view of a series of recent results, the inflammatory process should be reexamined to understand the potential and the beneficial effects of this process [3]. Altogether, these studies pave the way for a better understanding of the initial molecular and cellular events leading to pulp repair, as well as the development of the ideal materials to promote pulpal healing [3]. Partial pulpotomy after limited pulp capping, or total pulpotomy (namely, in deciduous teeth), and direct or indirect pulpectomy in permanently immature or older teeth constitute a whole range of clinical options [4]. The effects of Ca(OH)_2_ containing pulp capping agents on pulp cell migration, proliferation, and differentiation have been specified [5]. Ca(OH)_2_ induces beneficial effects due to chemical injury caused by the hydroxyl ions. A limited necrosis is induced against the vital pulp tissue (Figure 2). Necrosis provokes a slight irritation and stimulates pulp repair. Vascular and inflammatory cell migration and proliferation control mesenchymal and endothelial pulp cells, and also the formation of collagen (Figure 3). Odontoblasts differentiate and contribute to the formation and mineralization of a reparative dentinal bridge. Dentinal bridge develops following direct pulp capping. Tunnel defects favor the diffusion of bacteria issued from the oral cavity, which penetrate into the pulp (Figure 4). They contribute to microbial recontamination due to the numerous osteoblasts present in the reparative osteodentin bridge [6]. Inflammation of the tooth has been considered mostly as a negative factor leading to pulp destruction by necrosis or apoptosis. In short-term experiments, 1, 3, or 7 days after amelogenin implantation (A + 4 or A − 4), Osteopontin (OPN), which is both a matrix structural molecule and an inflammatory marker was gradually increased in the A + 4 implanted pulps. At 7 days, OPN expression began to decrease [7]. For later periods of time, OPN was used exclusively as a bone cell marker because no inflammatory reaction was detected. The labeling was roughly parallel with what was observed by using a RP59 antibody, which is a marker of bone marrow cells, primitive mesenchymal cells, erythroid cells, megakaryocytes, hematopoietic precursor cells, and osteo/odontoblast progenitors. Therefore, after an initial inflammatory burst, the committed cells underwent differentiation toward an osteoblast-like phenotype. The normal dental pulp contains heterogeneous cell population, including a majority of fibroblast-like cells, but also inflammatory and immune cells [dendritic cells (DCs), histiocytes/macrophages, T-lymphocytes], and also latent or dormant pulpal stem cells (progenitors), which are mostly involved in self-renewal (Figure 5) [3]. In the intact pulp, two distinct DC populations have been identified. CD11c^+^ are present at the pulp-dentin border, beneath occlusal fissures, whereas F4/80^+^ DCs are almost concentrated in the perivascular region of the inner pulp and in the subodontoblastic layer. CD11c^+^ dendritic cells express Toll-like receptors 2 and 4 and are CD205 positive. F4/80^+^ migrate from the inner pulp, increase in size, and display CD86 expression. Anti-inflammatory agents, including steroids, interleukin-1 (IL-1) receptor antagonist, soluble tumor necrosis factor (TNF) receptor, IL-10, nitric oxide (NO), heme oxygenase-1, and regulatory T lymphocytes (Tregs), are produced in order to limit tissue damage [8]. Pulp inflammation resulting from carious lesions is characterized by a strong increase in the production of proinflammatory cytokines, including TNF-*α*, IFN-*γ*, IL-1*β*, IL-6, CXCL8, and IL-18. IL-10, a cytokine that plays a central role in limiting host immune response to pathogens by promoting the development of Tregs, which are also upregulated.

Under deep caries, it is difficult to determine if the pulp is still alive or not, after bacterial invasion. Is it still possible to maintain the pulp tissue alive in the tooth? Facing an alternate possibility, the pulp should be partially or totally removed. Factors inducing inflammation may be spontaneously resolved, and in such case the pulp becomes fibrotic. It is also possible that mineralization is initiated at the periphery of the pulp, inducing the formation of a* reactionary dentin *very similar to bone or a bone-like tissue (Figure 3). In such case,* reparative dentin* may be formed, occluding the pulp exposure. It is also possible to observe diffuse mineralization or pulp stones limited in size within the pulp (Figure 4).

### 2.2. Necrosis

Affected pulp cells are recognized to die from two major processes: apoptosis and necrosis. It is now recognized that it is an oversimplification. Necrosis is a passive process due to the loss of protein functions or plasma membrane integrity [9]. Necrosis is caused by catastrophic toxic or traumatic events by passive cell swelling. The injury to cytoplasmic organelles, including mitochondria, leads to the rapid collapse of internal homeostasis (Figure 5). Leist et al. [10] have previously shown that intracellular energy levels are dissipated in necrosis, but not in apoptosis. Necrosis points membrane lysis, combined with the release of cellular contents that are implicated in inflammatory processes. Macrophages secrete cachectin and TNF. Bone resorption is stimulated and the phenomenon is occurring concomitantly with bone formation inhibition. Large zones of coagulation initially cause necrosis in contact with pulp connective tissue. Partially or totally infected dental pulps produce pulp calcification. A necrotic layer initiates revascularization and the construction of a dentinal bridge. Several small molecules and proteins are normally confined within cells. They are detected by specific receptors that induce a response characterized by the classical signs of inflammation at the tissue level. In areas of extensive cell death, tissue necrosis, also called coagulation necrosis, occurs.

Several other forms of cell death have been described. Cell death has been classified according to its morphological appearance (which may be apoptotic, necrotic autophagic, or associated with mitosis), functional aspects (programmed or accidental, physiological, or pathological), enzymological (with and without the involvement of nucleases or of distinct classes of proteases, such as caspases, calpains, cathepsins, and transglutaminases), or immunological characteristics (immunogenic or nonimmunogenic) [11]. Pyroptotic cell death has been described as a particular form of cell death in macrophage, induced by bacterial infection. It is accompanied by caspase-1 activation and the release of ILs. Pyroptotic cells may constitute defense mechanisms against microbial infection. The comparison between apoptosis, pyroptosis, and oncosis reveals important differences, which are shown in Table 1 [12, 13].

Caspases exist in inactive proforms in the cytosol and are activated by proteolytic cleavage by other caspases [14]. Caspases are broadly classified as apoptotic (Caspases 2, 3, 6, 7, 8, 9, and 10) or inflammatory (Caspases 1, 4, 5, and 12). Both pyroptosis and apoptosis are forms of programmed cell death that require specific caspase activity. Unlike apoptosis, pyroptosis occurs after caspase-1 activation, which does not involve in apoptotic caspases. Thus, apoptosis and pyroptosis are distinct forms of programmed cell death. Pyroptosis is viewed as a physiologically important form of cell death, which serves to eject intracellular pathogens from their replicative niche within macrophages.

### 2.3. Apoptosis

**Figure 6:**
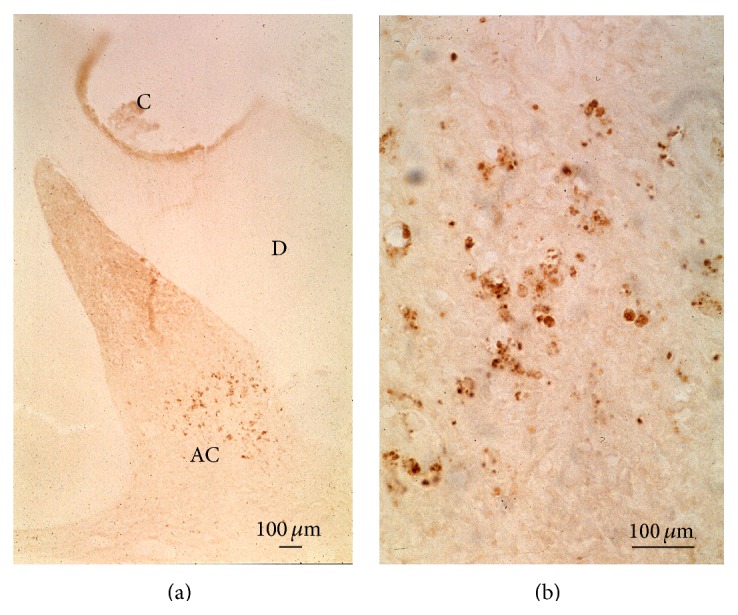
Apoptotic cells formation. After treatment of rat molar with a glass ionomer, apoptosis is visualized using the TUNEL method. (a) The reaction occurs some distance away inside the mesial pulp horn of a maxillary molar. C = cavity. Apoptotic cells (AC) accumulate in the pulp. (b) Higher magnification of apoptotic cells. Apoptotic cell nuclei are fragmented. Bar = 100 *μ*m.

Apoptosis or programed cell death is an active process, stimulated by environmental factors [9]. Apoptosis is characterized by cell shrinkage, membrane blebbing, leading to the formation of apoptotic bodies, and, if a nucleus is present, nuclear pyknosis, chromatin condensation, and genomic fragmentation (Figure 6).

Kitamura et al. [15] have shown that the c-jun N-terminal kinase (JNK) and heat-shock proteins (HSPs) are involved in apoptosis. JNK, c-Jun, and antiapoptotic HSP are expressed in a few pulp cells. HSPs were detected in the nuclei of pulp cells and relocalized from nuclei to the cytoplasm. Investigating the dental pulp by the TUNEL method, it was possible to show that some pulp cells display apoptosis, occurring in the dental pulp, but not in the odontoblast/subodontoblast layer [16].

### 2.4. Nemosis

Fibroblasts produce a significant amount of proinflammatory cytokines and cyclooxygenase-2 (COX-2). The process is characterized as programmed necrosis-like death, which has been named “nemosis.” Apoptosis is executed by caspase proteases, especially caspase-3. Although no activation of caspase-3 has been detected in nemosis, caspase inhibitors such as the pan-caspase inhibitor Z-VAD-FMK and the caspase-3 inhibitor Z-DEVD-FMK inhibited cell death by 40% and 80%, respectively [17].

Dental pulp inflammation may be a negative factor leading to pulp disruption [1]. The following three questions arise: (1) is the inflammatory reaction a prerequisite for the burst of progenitors implicated in pulp repair? (2) Does human dental pulp fibroblasts (HDPFs) formation lead in nemosis? (3) Does the adhesion between HDPFs lead to necrosis? In this context, it is well known that HDPFs express COX-2 and release prostaglandin E2 and IL-8.

**Figure 7:**
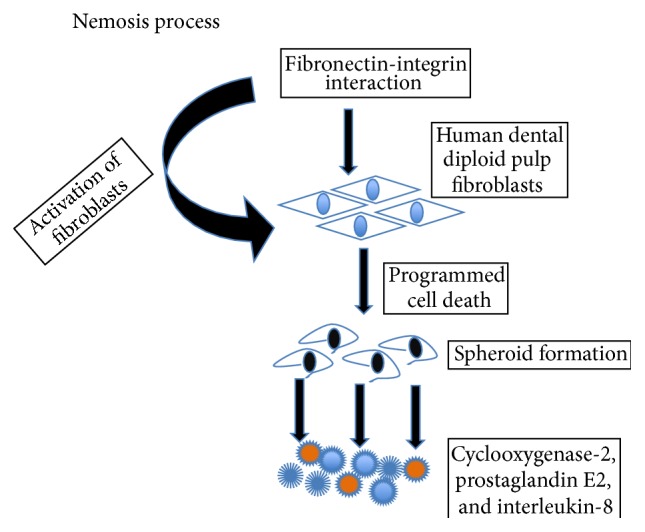
Illustration of nemosis process. Direct cell-cell interactions between diploid fibroblasts induce cell activation leading to programmed cell death. Nemosis of fibroblast generates large amounts of mediators of inflammation, such as prostaglandins, as well as growth factors. Factors secreted by nemotic fibroblasts also break down the extracellular matrix. Such factors include several MMPs and plasminogen activation.

Cells form spheroids were forced to cluster (also named spheroid formation). They are activated, leading to massive proinflammatory, proteolytic, and growth factor responses. Initiated by fibronectin-integrin interaction, the activation of fibroblasts ends in programming necrosis-like cell death (Figure 7).

Inflammatory reaction might be a prerequisite for the burst of progenitors implicated in pulp repair [1, 2]. Human dental pulp stem cells in culture constitute a model for* in vitro *nemosis-induced inflammation. HDPFs spheroid formation leads to necrosis. In response to nemosis, cell death is accompanied by the release of cyclooxygenase-2 and prostaglandin E2. The model supports that spheroids and interactions between fibroblasts and nemosis-targeted stem cells may contribute to treat pulp inflammation. Nemosis occurs in pulpal fibroblasts. Cell migrations may also be determined in cell sliding and migration from the root to pulp chamber [2]. Recently, it has been reported that apical stem cell niches are implicated in the sliding of apical cells from the end of the root (apex) toward the crown, where differentiation of progenitors may take place [18].

## 3. Apical Pulp Cells, Reparative Dentinogenesis, and Inflammatory Processes

### 3.1. Apexogenesis and Apexification

In young teeth, pulp vitality allows sustained root development, lengthening, and narrowing of pulp diameter. This is also named* apexogenesis* or* rhizogenesis*. If the pulp is irreversibly inflamed or necrotic, when the apex is not fully formed, procedures for the closure of apical foramina are required (*apexification*) [18, 19]. Hence, inflammatory processes in the root lead to formation three different dentins: (1) the development and lengthening of the root, (2) the formation of reactionary dentin along the root canal lumen, and (3) reparative dentin formation in the crown. In addition to the construction of the root, cellular cementum may be formed in the apical third of the root, leading to apical closure. Lateral, secondary and accessory canals contribute to the formation of complex arborescent structures. The cementum cap at the end of the root formation is apparently homogeneous and contributes to figure out how a root extension is formed. Reactionary dentin is formed, following indirect pulp capping with calcium releasing cements. Reactionary dentin is located beneath a calciotraumatic line. This newly formed dentin appears either as tubular (orthodentin structure), or as atubular with a bone-like appearance (similar to a osteodentin-like structure). It contains trapped osteocytes within osteoblasts lacunae, linked by thin canaliculi that are creating an interconnected osteocyte network. When the pulp is exposed, after capping the pulp surface is damaged by chemical injury, odontoblast-like cells differentiate beneath the scar. They polarize and are implicated in the formation of reparative dentin. This dentin, either tubular or atubular, again contributes to the formation of a dentinal bridge. The dentinal bridge is homogeneous or contains cell debris. It is also possible to have communication between the cavity and the superficial part of the pulp* via* tunnels that are containing pulp remnants. These tunnels favor bacteria communication and recontamination. More than likely, this is the reason of the failure of pulp capping after a short period of time. Tunnel and other defective structures may provide a pathway within reparative dentin for the penetration of microorganisms [6]. They are committed to develop secondary infection in pulp tissue.

Dentin phosphophoryn (DPP)/collagen composite was much superior to what resulted from calcium hydroxide regarding reparative dentin formation. Also, DPP/collagen composite displayed high ability in covering exposed pulp. It also induces the differentiation of human mesenchymal cells into odontoblast-like cells [20].

*Immunocompetent cells* are recruited in the pulp of rat after pulpotomy [21]. These cells are included within a population containing monocytes, macrophages, and stem/progenitor cells [22].

- * Monocytes and macrophage* originate from a common myeloid precursor in the bone marrow, expressing the colony-stimulating factor-1. The life of blood monocytes lasts just a few days before undergoing apoptosis. Monocytes can switch from a short live, undergoing apoptosis within a day to a prolonged survival during inflammation. They may go back quickly to a short live when the inflammation resolves. Macrophage life may expand up to a couple of months. Macrophages' life span has less plasticity. They may live longer and are quite resistant to apoptotic stimuli. Differentiation and inflammation determine monocyte/macrophage lifespan, by blocking the apoptotic pathway and activating many survival pathways.

There are many monocytes subpopulations, acting differently during pathogen recognition. The granulocytes are normally between 5,000 and 10,000 cells/mm^3^ and they are composed of neutrophils (50–70%), eosinophils (2–4%), and basophils (0.5–1%). The agranulocytes are composed of lymphocytes (20–40%) and monocytes (3–8%), totally 300–700 cells/mm^3^. The leukocytes play a fundamental role in the immune system by responding to a diverse repertoire of pathogens, including bacteria, viruses, and parasitic and fungal infections, and also in some pathological conditions against the host cells. Monocytes and macrophages are components of the innate immune system that are responsible (1) for the recognition of the inflammatory stimuli, (2) the initiation of the inflammatory response that is characterized by the production of proinflammatory cytokines, and (3) the clearance of the pathogens allowing the resolution of inflammation. It is recognized that defined surface expression molecules characterize specific subpopulations of monocytes. They constitute the main source of resident or recruited tissue macrophages found at sites of inflammation.* Macrophages* infiltrated wound-healing sites between 1 and 28 days. Initially macrophages were described as large phagocytic cells having the capacity of “eating” wounded cells. ED1+ (CD68+) increased throughout the root pulp during an inflammatory phase. OX6+ macrophage that expresses class II MHC increases in the pulp and declines thereafter. OX6+ cells appear prior to dentin bridge formation and continue to appear during the healing stage at 14 days.

The process of differentiation from monocyte to macrophage is initiated once monocytes reach the target tissue. Monocytes can differentiate into tissue macrophages, DCs, and osteoclasts. The patrolling behavior of monocytes and macrophages is essential in the initial host response to infection. The initiation and resolution of acute and chronic inflammation are mediated by the activation of monocytes and macrophages, which are triggered by the recognition and phagocytosis of pathogens through specialized receptors.

(ii) * Stem/progenitor cells* have been identified in normal and inflamed pulp [23]. The term “stem cells” includes pluripotent cells that have an unlimited capacity to divide and are specifically adapted for permanent survival. Therefore, the next question relates to dental pulp stem cells (DPSCs) and whether they exist in the inflamed pulps (IPs). The comparison between normal and inflamed pulp cells opens in the next question. We wonder if IPs are present at higher levels in mesenchymal pulp where stem cell markers are found, such as STRO1, CD90, CD105, and CD146, or if they are present at low levels.

Leprince et al. [24] concluded that isolation and characterization of mesenchymal stem cells (MSCs) are essential for dental pulp repair. Of note, bone marrow-derived MSCs and DPSCs are probably the same, or at least of the same family. DPSCs may be recruited and be crucial for the success of regenerative endodontic procedures. The expression of specific surface antigens, for example, CD29, CD73, CD90, and CD105, may be typical for MSCs. This labeling is parallel with the absence of other surface antigens, such as CD34 or CD45. Immunocompetent cells, and especially T-lymphocytes (CD8+ T cells), differentiate into cytotoxic T cells and CD4+ T cells. They comprise also a collection of helper T cells, producing mainly Th1 and Th2. Th1 cells activate macrophages, which can produce various inflammatory mediators such as IL-1, platelet-activated factor, prostaglandins, and leukotrienes [25]. Bone marrow-derived MSCs express receptors for a large number of cytokines (e.g., IL-1, IL-4, IL-6, INF-*γ*, TNF-*α*), and growth factors [e.g., fibroblast growth factor (FGF), platelet-derived growth factor (PDGF), transforming growth factor-beta (TGF-*β*), epidermal growth factor (EGF), insulin-like growth factor (IGF), and bone morphogenetic proteins (BMPs)] and chemokines.

### 3.2. Pulp Capping Hydroxide

The mechanisms of most pulp capping hydroxide implicate that Ca(OH)_2_ has a high pH (alkalinity) producing superficial necrosis when placed directly on exposed pulp tissue [5]. The biomaterial stimulates mineralization (the formation of dentin-like hard tissue separating the pulp from the necrotic capping agent) and has antibacterial properties. Following pulp exposure and capping, early changes include hemorrhage and moderate inflammation, resolved during the first week. The presence of calcium ions stimulates the precipitation of calcium carbonate in the wound area and thereby contributes to the initiation of mineralization. Then, the differentiation of pulp cells occurs; cells are bearing the phenotypic characteristics of odontoblast-like cells and are forming a dentin-predentin-like collagen-rich matrix. Recently, mineral trioxide aggregate (MTA) has become a popular alternative for Ca(OH)_2_, which is composed of calcium oxide in the form of tricalcium silicate, dicalcium silicate, tricalcium aluminate, and bismuth oxide for radiopacity [26]. The MTA has a higher success rate and results in less pulpal inflammatory response and more predictable hard dentin bridge formation than Ca(OH)_2_ [27]. MTA appears to be a suitable replacement of Ca(OH)_2_ used for direct pulp capping; however Ca(OH)_2_ has been considered the “gold standard” of direct pulp capping materials for several decades [28, 29].

Dentin fragments, which are displaced into the pulp during cavity preparation, are acting as initial loci for mineralization or pulp stone formation [30, 31]. Pulp inflammation, which is developed following carious lesion, is characterized by a strong increase in the expression of proinflammatory cytokines, including TNF-*α*, IFN-*γ*, IL-1*β*, IL-6, CXCL8, and IL-18. Interestingly, IL-10 is a cytokine that plays a central role in limiting host immune response to pathogens and promotes the development of Tregs.

Necrosis causes slight irritation. It stimulates the defense and repair of the pulp. The observed sequence of tissue reactions exactly displayed what could be expected when a connective tissue is wounded. It starts with vascular and inflammatory cell migration, and proliferation, toward the final tissue control, eliminating the irritating agent. This process ends by the tissue repair, including migration and proliferation of mesenchymal and endothelial pulp cells, which is monitored by the formation of collagen. Human pulp in culture expresses various growth factors and cytokines, implicated in the syntheses of DNA, type I collagen, laminin, fibronectin, osteonectin/SPARC protein, and alkaline phosphatase (ALPase) [32].

The levels of type I collagen and laminin per cell were remained almost constant after culturing human pulp cells. In contrast, secreted proteins that were acidic and rich in cysteine (SPARC/osteonectin) and ALPase levels were markedly increased when the cell cultures reached confluence. Laminin and type I collagen, as well as fibronectin, stimulate the spreading of pulp cells within 1 h [33]. At 28 days, fibronectin, which is implicated in the formation and mineralization of tubular dentin, participates in the differentiation of odontoblast-like cell [33]. The addition of TGF-*β* to the culture medium decreased laminin and ALPase levels, whereas it increased SPARC and fibronectin levels 3- to 10-fold [32]. Western and Northern blots showed that TGF-*β* enhanced SPARC synthesis at the protein and mRNA levels. Basic FGF (bFGF) decreased type I collagen, laminin, SPARC, and ALPase levels without changing the fibronectin level [32]. PDGF selectively decreased laminin, SPARC, and ALPase levels. EGF also decreased SPARC and ALPase levels. TNF-*α* and IL-1*β* decreased type I collagen and laminin levels and abolished SPARC and ALPase syntheses. bFGF and PDGF showed the greatest stimulation of [^3^H] thymidine incorporation into DNA [32]. TGF-*β*, EGF, and TNF-*α* had less effect on DNA synthesis, whereas IL-1*β* inhibited DNA synthesis.

Altogether, these findings demonstrated that TGF-*β*, bFGF, EGF, PDGF, TNF-*α*, and IL-1*β* have characteristically different patterns of actions on the DNA, laminin, type I collagen, fibronectin, ALPase, and SPARC syntheses by pulp cells [32]. However, there is also an alternative possibility. Noninflammatory processes may be implicated in cell recruitment, proliferation, and differentiation of pulp cells expressing phosphorylation/mineralization proteins of the extracellular matrix. There are some indications that bacteria may differently affect the odontoblasts' ability to repair the dentine barrier [34]. From the published data, there are some examples of capping effects without being associated with inflammatory processes [34].

*Emdogain Gel* initiated dentine formation, though not in a form that could constitute a solid barrier [34]. There is no evidence showing that an increased pH, and simultaneously a chemical injury, limited necrosis. The release of FGF2 delivered by a collagen sponge (noncontrolled release) or incorporated in gelatin hydrogel (controlled release) stimulates the migration and proliferation of pulp cells, followed by the invasion of vessels into dentin defects. The noncontrolled release of free FGF2 from collagen sponge induced excessive reparative dentin formation in the residual dental pulp. In contrast, controlled release of FGF2 from gelatin hydrogels induced the formation of dentin-like particles with dentin defects above exposed pulp [35].

Hindering the penetration of proinflammatory cells and/or cytokines enhanced the viability of MSCs. Using anti-inflammatory drugs and an alginate hydrogel scaffold, a molecular and cellular based investigation was reported to improve the application of hydrogels in stem cell-based therapies [36]. Along the same lines of evidences, we have implanted a light-cured hydrogel based on bovine serum albumin and glutaraldehyde within the exposed pulps of rat molars. Implanted after one week, inflammation was much more moderate compared to Dycal capping. Gradually, reparative dentin was formed, closing the pulp exposure. Dentinal bridges were formed after 3 weeks following Dycal-capping. They display tunnels and osteoblast lacunae containing osteocyte-like cells (osteodentin). The closure of the pulp exposure was not completed, and more than 4 weeks were needed to fill the gap. In contrast, the hydrogel formed more expanded and homogenous reparative dentin. There was no evidence for inflammatory processes. In both situations, active formation of the reactionary dentin layer was induced; however Hydrogel contributed more extensive dentin layer comparatively to Dycal. Hydrogel is acting as a biodegradable cavity liner, which is based on cross-linked proteinaceous material of animal/human origin [37].

The reactionary-forming dentin is not linked to pulp exposure and inflammation. There are also two different pathways of forming reparative dentin after pulp exposure. Two reparative dentins (osteodentin and orthodentin) are resulting from pulp exposure associated with inflammation or noninflammatory pulp exposure processes.

### 3.3. Expression of Extracellular Matrix Proteins and Pulp Inflammation

Phosphorylated extracellular matrix proteins (SIBLINGs) are synthesized by odontoblasts and subjacent to Hoehl's cells of dental pulp. These proteins may contribute to pulp repair and regeneration efficiently. The roles of SIBLINGs and MMPs are not diverging. SIBLINGs, as most of the phosphorylated extracellular matrix proteins, are related to the mineralization process and hence are instrumental in pulp regeneration once the inflammatory process is resolved. By contrast, MMPs are related to catalytic processes. They are acting on procollagen chains fibrillation, contributing to the cleavage of the amino and carboxyl propeptides. In addition, MMPs play role in the cleavage of dentin sialophosphoprotein (DSPP) into dentin sialoprotein and DPP. Therefore the two molecules contribute in this cascade of events reducing firstly the inflammation process, and afterwards, acting as an effective regeneration agent. In an experimental approach using germ-free and conventional laboratory rats, Kakehashi et al. [38] showed enhanced pulp repair in germ-free rats. The absence of microbial flora was the major determinant for the healing of exposed rodent pulp. They concluded that the absence of infection and inflammation was essential for tissue healing. SIBLINGs and MMPs contribute, respectively, to resolve inflammatory processes and stimulate regeneration processes.

#### 3.3.1. SIBLINGs and Pulp Repair

*DSPP* expression is high in human dental pulp [39]. DSPP plays a role in the dentin mineralization process and is also implicated in the immune response. Leptin, an inflammation-related adipokine, and its receptor (LEPR) are expressed by human dental pulp. Immunoblot analysis and RT-PCR showed that DSPP are concentrated over the odontoblast layer; however, their presence is questionable in the central zone of the pulp.* Bone sialoprotein* produces slight inflammation following implantation to coronal pulp, and it is implicated in the formation of a homogeneous atubular dentin-like structure in the mesial part of the coronal pulp chamber one month later implantation. OP-1 (BMP7) induces the formation of osteodentin in the coronal pulp, in contrast with the radicular part of the pulp totally filled by a mineralized material after OP1 implantation [40]. According to Abd-Elmeguid et al. [41]* osteocalcin* (OCN) is a reparative molecule expressed inside the dental pulp and involved in pulpal inflammation. OCN was positively correlated with the expression of vascular endothelial growth factor, FGF, macrophage inflammatory protein-1*β*, monocyte-derived chemokine, monocyte chemoattractant protein-1, IL-17, and soluble IL-2 receptor *α*. It was negatively correlated with that of IL-1*α*, IL-1*β*, IL-8, granulocyte macrophage colony-stimulating factor, and macrophage inflammatory protein-1*α*. Altogether, these different properties are leading to new molecular treatment strategies. Following the role of phosphorylated proteins, Abd-Elmeguid et al. [41] has shown that dentin matrix protein-1 (DMP-1) is mostly localized in the inflammatory crown, but lacking in root pulp. DMP-1 stimulates the production of IL-6, IL-8 and has an additive effect on the release of bacterial lipopolysaccharide (LPS) on pulp cells [42].

#### 3.3.2. Matrix Metalloproteinases (MMPs)

MMPs are a large family of calcium-dependent zinc-containing endopeptidases, which are responsible for the tissue remodeling and degradation of the ECM, including collagens, elastins, gelatin, matrix glycoproteins, and proteoglycan. MMPs are excreted by a variety of connective tissue and proinflammatory cells including fibroblasts, osteoblasts, endothelial cells, macrophages, neutrophils, and lymphocytes.

Expression of MMP-3 was upgraded at 12 and 24 h after pulp injury, whereas MMP-2 and MMP-14 were not changed [43]. MMP-3 was localized in endothelial cells or endothelial progenitor cells in injured* in vivo* pulp. MMP-3 enhances cell proliferation, migration, and survival. It induces angiogenesis and reparative dentin formation. In isolated pulp-derived CD31−, CD146− side population cells have a highly vasculogenic potential. MMP-3 was highly expressed in CD31−, CD146− side population cells compared with CD31+, CD146− side population cells, which are without vasculogenic potential. Both MMP-9 and MMP-2 were weakly expressed in cell fractions. When CD31−, CD146− side population cells were transplanted on the amputated pulp in dogs, the transplanted cells were migrating in the vicinity of the newly formed vasculature and expressed proangiogenic factors, including MMP-3, implying trophic actions on endothelial cells [44].

#### 3.3.3. Other Molecules

*Mediators*. Wnt5a is involved in inflammation regulation. Wnt5a was increased 9-fold in human dental pulp cells (HDPCs) after TNF-*α* stimulation compared with control cells [45]. HDPCs treated with Wnt5a or its supernatant increase macrophage migration (recruitment and inflammatory mediator in human pulp inflammation). Wnt5a is mitogen-activated protein kinase (MAPK) dependent and NF-*κ*B dependent. Wnt5a is an inflammatory mediator driving the integration of cytokines and chemokines, acting downstream of TNF-*α*. Considering how pulpitis drives tissue destruction, an important step in supporting the regeneration of pulpal tissues is the attenuation of inflammation. Macrophages, key mediators of the immune response, may play a critical role in the resolution of pulpitis due to their ability to switch to a proresolution phenotype. This process can be driven by the resolvins, a family of molecules derived from fatty acids that show great promise as “therapeutic agents” [46]. Controlling inflammation facilitates dental pulp regeneration. Macrophages and neutrophils are mediators of the innate inflammatory response in the dental pulp. B and T cells of the acquired immune system infiltrate the pulp and contribute to the inflammatory response, releasing proinflammatory cytokines, IL-1*α*, IL-1*β* and TNF-*α*, and MMPs. Two broad classes of resolvins have been characterized: the E- and the D-series, which are derived from eicosapentaenoic acid and from docosahexaenoic acid, respectively [47, 48]. Resolvins exert numerous potent anti-inflammatory effects, such as decreasing the migration and activation of neutrophils [49–51]. They inhibit the production of IL-12 by dendritic cells [52] and enhance the appearance of M2 phenotype pro-resolving macrophages [52, 53]. Resolvin E1 (RvE1) has shown efficacy in a dental context. RvE1 acts to downregulate NF-*κ*B through the ligand specific receptor Chem R23, which is expressed by a number of cell types, including monocytes/macrophages, neutrophils, dendritic cells, and T cells [52, 54].

#### 3.3.4. Vascularization

Dental pulp is encased in dentin, which plays a role as a barrier against bacterial, chemical, and physical stimuli. When the barrier is disrupted by traumatic injury or caries, the dentin-pulp complex has a potential to repair and regenerate. Angiogenesis is essential for this pulp wound-healing process, because blood vessels play an important role in nutrition and oxygen supply, as a conduit for transport of metabolic waste, pulp homeostasis and metabolism, and stem/progenitor cell migration [55]. During pulp wound-healing process, dental pulp stem/progenitor cells migrate to the injured site from perivascular region in the pulp tissue deeper from the injured site [55]. They proliferate and differentiate into endothelial cells for angiogenesis/vasculogenesis or into odontoblasts for reparative dentin formation [56]. The angiogenic signals, such as vascular endothelial growth factor (VEGF), bFGF, and TGF-*β*, released from injured dental pulp cells, endothelial cells, and ECM by injury contribute to the migration of stem/progenitor cells [57–59]. In the inflamed dental pulp emphasis was put on the enlargement of blood vessels, the VEGF labeling, and pericytes, which might be at the origin of endothelial cells. The presence of enlarged vessels indicates (1) an increased tissue fluid pressure; (2) a greater outward flow of dentinal fluid; and thus (3) an increased pain after dentinal stimulation [60]. Neoangiogenesis is a requirement for regeneration and healing, highly controlled by the microenvironment [61]. Precise mechanism for migrating stem/progenitor cells and angiogenesis/vasculogenesis during pulp wound-healing process, however, still remains unclear.

## 4. Conclusion

Different phases of pulp inflammation have been identified, associated with moderate inflammation, necrosis including pyroptosis, apoptosis, and nemosis. For many years the importance of inflammation in pulpal healing has been underestimated, considered only as an undesirable effect. There are now evidences that inflammation is a prerequisite for tissue healing and pulp regeneration. Immunocompetent cells (monocytes, macrophages, and stem/progenitor cells) are recruited. Cells slide along the root and migrate toward the crown. Due to the high alkalinity of the capping agent, mineralization is initiated and becomes thicker. Due to inflammatory processes, pulp cells proliferate and display increased number and size. Their phenotype is modified, and they become odontoblast-like cells producing collagen, ALPase and SPARC/osteonectin.

Molecules of the SIBLING family, MMPs, mediators, and scaffolds are also implicated in the formation of a reparative dentinal bridge. These molecules are implicated in the direct formation of osteo/orthodentin, occluding the pulp exposure. There is also an alternative possibility that noninflammatory processes contribute to produce reparative dentin. This suggests that there is occurrence of different reparative pathways after the pulp exposure. Dentins are formed as reactionary dentin, an accumulation occurring at the surface of the dental pulp, along the periphery of the pulp chamber, or as reparative dentin creating a dentinal bridge occluding partially or totally a pulp exposure.

## Figures and Tables

**Table 1:** The comparison of cellular events between apoptosis, pyroptosis, and oncosis.

	Apoptosis	Pyroptosis	Oncosis
Initiating	Programmed	Programmed	Nonprogrammed, accidental
Signaling pathway	Caspase-3/6/7, DNA fragmentation	Caspase-1 DNA fragmentation	Noncaspase
Terminal event	Nonlytic, plasma membrane blebbing	Lytic, pore formation, and release of cytokines	Lytic
Effect on tissue	Noninflammatory, formation of apoptotic bodies	Inflammatory	Inflammatory
Cell types	All	Macrophages and DCs	All
